# The effectiveness of an enhanced invitation letter on uptake of National Health Service Health Checks in primary care: a pragmatic quasi-randomised controlled trial

**DOI:** 10.1186/s12875-016-0426-y

**Published:** 2016-03-24

**Authors:** Anna Sallis, Amanda Bunten, Annabelle Bonus, Andrew James, Tim Chadborn, Daniel Berry

**Affiliations:** Public Health England, 2nd Floor Skipton house, 80 London Road, London, SE1 6LH UK; Department of Health, 5th Floor Richmond House, 79 Whitehall, London, SW1A 2NS UK

**Keywords:** NHS Health Check, Cardiovascular disease, General practice, Primary prevention, Implementation intentions, Simplification, Behaviour change techniques, Behavioural insights, Prompts

## Abstract

**Background:**

The National Health Service Health Check (NHS HC) is a population level public health programme. It is a primary prevention initiative offering cardiovascular risk assessment and management for adults aged 40–74 years (every five years). It was designed to reduce the incidence of major vascular disease events by preventing or delaying the onset of diabetes, heart and kidney disease, stroke and vascular dementia . Effectiveness of the programme has been modelled on a national uptake of 75 % however in 2012/13 uptake, nationally, was 49 %. Ensuring a high percentage of those offered an NHS HC actually receive one is key to optimising the clinical and cost effectiveness of the programme.

**Methods:**

A pragmatic quasi-randomised controlled trial was conducted in four general practitioner practices in Medway, England with randomisation of 3511 patients. The aim was to compare attendance at the NHS HC using the standard national invitation template letter (control) compared to an enhanced invitation letter using insights from behavioural science (intervention). The intervention letter includes i) simplification - reducing letter content for less effortful processing ii) behavioural instruction - action focused language iii) personal salience - appointment due rather than invited and iv) addressing implementation intentions with a tear off slip to record the date, time and location of the appointment. Logistic Regression explored the association between control and intervention group and attendance at a health check.

**Results:**

29.3 % of patients who received the control letter and 33.5 % of those who received the intervention letter attended their NHS HC (adjusted odds ratio 1.26, 95 % confidence interval 1.09–1.47, *p* < 0.01). This was an absolute difference in uptake of 4.2 percentage points for those receiving the intervention letter.

**Conclusions:**

An invitation letter applying behavioural insights was more effective than the existing national template letter at encouraging attendance at an NHS HC. Making small, no cost behaviourally informed changes to letter invitations can improve uptake of the NHS HC. Further research is required to replicate the effect with more robust methodology and powered for sub-group analysis including socio-economic status.

**Trial Registration:**

Current Controlled Trials ISRCTN66757664, date of registration 28/3/2014.

**Electronic supplementary material:**

The online version of this article (doi:10.1186/s12875-016-0426-y) contains supplementary material, which is available to authorized users.

## Background

Reducing avoidable premature mortality is a government priority. In 2009, the Department of Health introduced a phased implementation of the National Health Service Health Check (NHS HC) programme in England. It uses a preventative population-based approach, involving a cardiovascular risk assessment and management programme for adults aged 40–74 years. It was designed to reduce the incidence of major vascular disease events by preventing or delaying the onset of diabetes, heart and kidney disease, stroke and certain types of dementia [1]. Everyone aged 40 to 74 years (who has not already been diagnosed with one of these conditions or who has certain risk factors) should be invited once every five years for a risk assessment and then given lifestyle support and advice to reduce or manage their risk. Routine health tests are performed as part of the risk assessment including a cholesterol test that requires a blood sample.

The NHS HC is a national programme, delivered by local arrangements to fit local context ensuring equity of access [2]. The format of the NHS HC may vary depending on where it takes place but in most cases it consists of a face to face individual risk assessment with a trained health professional. The discussion of the results and lifestyle support and advice may happen at the same appointment or at a later date when the results are available.

The provision of NHS HC risk assessments is a mandatory requirement for Local Authorities (LA) [3]. LAs have flexibility on who they commission to provide the service and what locations are used to deliver the check but it was predominantly delivered through primary care in 2013. The tests and measurements however, are standardised to help ensure the safety, quality and effectiveness of the programme. It is also key that the actions taken at certain thresholds are the same, to assure a systematic and uniform offer across England and to maximise the public health impact of the programme. Beyond this, there is considerable variation in delivery across LAs.

LAs must achieve a 100 % offer rate in their eligible populations after five years of the programme start date, which means this target needed to be reached by 2013. Ideally LAs will offer the NHS HC to 20 % of their eligible population each year, reaching 100 % over the five years from 2009. Across England, there are approximately 15 million people in this age group who should be offered an NHS HC once every five years [4]. Funding has been allocated to support this scenario and is modelled on an uptake rate of 75 %. However in 2011/12, the NHS Health Check had lower median coverage of 8.2 % compared with the anticipated 18 % coverage by this date [5]. LAs have a legal duty to seek continuous improvement in the percentage of eligible individuals taking up their offer of an NHS HC as part of their statutory duties. Ensuring a high percentage of those offered an NHS HC actually receive one is key to optimising the clinical and cost effectiveness of the programme [6]. This is especially important for populations with the greatest health needs. Yet, data show that there is considerable variation in offers and uptake across LAs ranging from 0 to 29.8 % coverage [5].

Currently there are no set targets on uptake however guidance states areas should aspire to take up rates comparable with other screening programmes which achieve around 75 % [1]. Learning from similar programmes has demonstrated that it takes time to increase uptake rates and with the programme still in its early stages, it is encouraging that the national take-up rate in 2011/2012 was 52 %, although dropped slightly in 2012/13 to 49 %. There is however, a significant drop off rate from those invited to attend an NHS HC, the number who respond, attendance and treatment uptake. A study on the uptake of the NHS HC in Stoke on Trent showed 63.3 % of those invited to a health check responded, 43.7 % attended a check and 29.8 % of those who needed to took up treatment [7]. In 2013, 14,814 people were invited to an NHS HC in Medway but only 31 % attended.

Few countries have introduced large scale cardiovascular risk assessment programmes and the evidence of increasing uptake in routine care settings is sparse [8]. Although recently Forster et al. published a study protocol to test the hypothesis that enhanced invitation methods using the question behaviour effect will increase uptake of the NHS HC compared with a standard invitation [9]. There is a need to ensure high quality research is conducted to ensure the programme is being delivered effectively and delivers its aims as a population level primary prevention programme.

### Invitation process

A range of different invitation processes and services have been commissioned from providers and delivered by a range of health care professionals. NHS HCs are offered to the eligible population either by a letter invitation or opportunistically. Opportunistic invitations typically occur through community outreach, community based events or within practices and work places. They may be verbal or through a marketing campaign offering either immediate access to a health check or directing them to their local provider. Face to face recruitment can have advantages for socio-economically disadvantaged populations by enabling greater exchange of information about the importance and relevance of health screening and prevention [10].

Literature has shown response rates to invitations to attend medical screening vary by condition, ethnicity, and are notably lower among socially deprived groups [11]. A Cochrane Review of interventions to encourage the uptake of cervical screening showed there is evidence to support the use of invitation letters [12]. Invitation via letters from a patient’s GP practice is the most common route for inviting the eligible population for an NHS HC. A national template was developed following qualitative research, but there is no robust evidence on its effectiveness. Therefore this study was designed to test optimisation of the invitation letter as a potentially low cost and scalable way to improve uptake and overcome some of the previously reported issues with appointment letters such as letters being ignored or forgotten, if indeed they are recalled as having been received at all [13].

Uptake of NHS Health Checks has also been seen to vary by socio-demographic characteristics of the invitees. Two studies in West London found that uptake was significantly higher among women and older patients [8, 14]. Dalton et al. also found that South Asian or mixed ethnic background adults were more likely to attend and that there was no difference in uptake by deprivation quintile of area of residence (although there were too few areas with the lowest levels of deprivation in the study setting to conclude the latter with confidence).

One reason why uptake is suboptimal for all of these services could be due to the intention-behaviour gap. The intention-behaviour gap describes self-regulatory problems with goal realisation whereby a goal is formed but the intended behaviour is not enacted. Forming an implementation intention can help to overcome this gap [15] and various techniques for doing so have been developed and tested across a range of behaviours from voting to vaccination. Milkman et al. [16] improved flu vaccination uptake amongst employees by 4.2 percentage points simply by prompting them to write down the date and time of their appointment. Another study by the same authors found that mailed reminders to employees including a sticky note to record the date and name of the doctor increased attendance at colonoscopies by a statistically significant difference of 1 percentage point or a 15 % difference relative to the control group [17]. Nickerson and Rogers used implementation intentions techniques to increase voter turnout using a telephone script to encourage registered voters to attend voting polling stations to cast their vote. The implementation intentions script asked voters what time they would vote, where they would be coming from and what they were doing beforehand. Combined with a self-prediction script they increased voter turnout by 4.1 percentage points [18].

Evidence from other areas suggests that making small changes to the wording of letters alone can have big impacts on behavioural responses. This was demonstrated in a trial to increase tax compliance. Letters from Her Majesty’s Revenue & Customs to taxpayers yet to pay their tax liabilities led to millions of pounds in increased tax payments [19]. Shah and Oppenheimer [20] also emphasise the importance of integrating less information to reduce the effort associated with tasks and subsequent impact on decision making. Therefore simplifying the information presented could increase the decision to engage with the NHS HC.

This study aimed to test the impact of an enhanced invitation letter on attendance at an NHS HC appointment compared to the standard national template letter.

## Methods

### Trial design and ethics

The study was a quasi-randomised controlled trial with one intervention arm. Ethical approval was received from the NHS National Research Ethics Service Committee North West - Greater Manchester East (NHS REC 13/NW/0399). Individual consent for participation was not sought as researchers only had access to anonymised data, no individual level data are presented, and because informed consent would likely have influenced individuals’ behaviour therefore affecting the ecological validity of the study and extrapolation of its findings. To protect confidentiality, anonymised, aggregate data can be obtained by contacting the authors.

### Study recruitment

Four GP practices in Medway were purposively selected due to having large numbers of patients eligible for NHS HCs in 2013/2014, suitable IT systems and centrally administered systems for distributing the letters. In Medway the NHS HC is offered to all patients aged 40–74. Each year, those turning 41, 46, 51, 56, 61, 66 or 74 on their next birthday are invited for an NHS HC. Invitations are sent in one batch, each year in May and June.

**Fig. 1:**
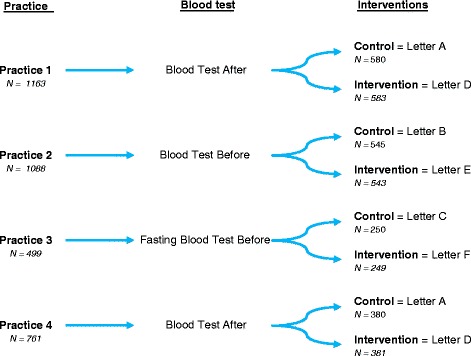
Trial Design: Number of patients invited per practice across control and intervention letters

The cholesterol blood test element of the NHS HC takes place at another appointment; after their NHS HC appointment in practices 1 and 2 and before the NHS HC in practice 4. Practice 3 invites patients to attend an appointment for a fasting blood test before the NHS HC appointment, requiring the patient to not eat or drink anything except water for eight to ten hours prior to the appointment (See Fig. 1.). Attendance is recorded if both the health check and blood test elements are attended.

### Procedure

A list of the 3511 patients eligible for an NHS HC in 2013/14 registered at one of the four practices was generated from patient records accessed by the Medway NHS HC manager. These were extracted into a database and ordered alphabetically. The IT systems in place for sending out the letters meant that it was not possible to truly randomly allocate the participants to the control or intervention letters. Instead, the local NHS HC manager allocated participants to control and intervention groups by surname divided at the midpoint within their practice lists. In order to minimise bias those with surnames in the first half of the list received the control letters at two practices and intervention letters at the other two primary care practices; and vice versa. Practice staff were unaware which intervention group participants were allocated. In May 2013, the NHS HCs manager mail-merged and printed the letters. All letters were posted to patients during May and June.

### Intervention

The letter sent to the control group was the existing invitation letter used in Medway, which was based on the national template [21]. The letter was the same across all practices apart from one paragraph which outlines the different arrangements for blood testing (example in Additional file 1).

The intervention letters [example in Additional file 2] involved four key changes. The first was simplification - the letter was shortened to two, one sentence paragraphs plus a headline call to action to improve readability and reduce complexity. This type of simplification was aimed at reducing the effort required for information processing, recognizing that humans are “cognitive misers” with a preference for simple over complex information [22]. King et al. for example, found making prescription forms simpler and clearer significantly reduced prescription errors [23]. Information removed from the letter was already contained in the associated information leaflet which patients were directed to in the letter and which was sent with all letters to ensure patients have the opportunity to make an informed choice about the benefits and risks of attending.

The second set of changes aimed to improve the behavioural specificity of the letter using a behavioural instruction and concrete statements. The action (calling to book an appointment) was made more prominent by using an instructional statement in bold type face in the first sentence of the letter. Michie and Johnston amended the language used in the National Institute for Clinical Excellence guidelines for the management of schizophrenia to improve behavioural specificity. In doing so they improved mental health service users attitudes towards, intentions to act and perceived behavioural control over guideline implementation compared to a control group reading the guidelines in place at the time. Their work was based on principles of plain English, behaviour change being more likely when specified in concrete terms [24] and specifying how, what, when, where and why being associated with improved comprehension and recall [25, 26].

Thirdly, to increase the personal salience of the letter the intervention letter contained an emboldened heading stating ‘you are due to attend your NHS Health Check’. The intention of using the word ‘due’ rather than ‘invited’ was to infer personal relevance of the letters timing (indicating the five year cycle for the NHS HC).

Finally by adding a tear-off slip with space for patients to record the date, time and location of their NHS HC with an instruction to stick it to their fridge, the letter intended to address the intention-behaviour gap, (see [27] for review) through planning prompts. Stating when, where and how one will undertake an action rehearses the cognitive link between behaviour and the context of performance. This aspect of the intervention was similar to Milkman et al. [19, 20] described earlier.

According to the Behaviour Change Techniques Taxonomy Version One (BCT-T V1) completing the tear off slip would be classified as ‘action planning’ BCT 1.4 and asking recipients to stick the slip to their fridge could be classified as ‘prompts/cues’ BCT 7.1 [28]. The authors were unable to categorise the other three changes using the BCT-T V1.

Intervention letters were the same for each practice besides the essential variation in attendance instructions due to the three different local blood test arrangements across the four practices. Therefore three different letter combinations were developed. The three control letters are labelled A, B and C and the three intervention letters are labelled D, E and F as described in Fig. 1.

### Outcome measure

The primary outcome was attendance at an NHS HC (binary measure).

### Power calculation

A power calculation was based on a provisional sample size of 3580 (1790 per arm) and an assumption that the existing attendance was at 40 %. Based on similar studies such as Milkman et al. [17], we estimated that the study could achieve a four to five percentage point difference. The study was therefore set to detect a 4.6 % difference in control and intervention groups for our primary outcome measure of attendance at an NHS HC, with 80 % power and 5 % significance.

### Statistical analysis

Data were analysed in SPSS (2011, version 20.0 [29]). In addition to the two trial arms (intervention and control), the explanatory variables were age, gender, deprivation quintile (categorised by patient postcode mapped to the Index of Multiple Deprivation; 1 = 20 % most deprived, 5 = 20 % least deprived), and GP practice (Table 1). Age in years was analysed as a continuous linear variable. The quasi-randomisation was checked using chi-squared tests and a one-sided t-test to check for differences between control and intervention groups by the other explanatory variables. Multivariable logistic regression was used to assess the independent contribution of the intervention (Attenders =1; non-attenders = 0) and protect against chance imbalance of the covariates. We additionally tested for an interaction between the intervention and the GP practice due to the differences in process described above (Table 1).

**Table 1:** Characteristics of patients invited to attend an NHS HCs by trial arm

		Control (*n* = 1755)	Intervention (*n* = 1756)	*P* values from chi^2^ and t-test
Gender [*n* (%)]	Male	816 (46.5)	862 (49.1)	0.13
	Female	939 (53.3)	894 (50.9)	
Age [mean (s.d.)]	Years	53.1 (9.76)	52.8 (9.78)	0.47
Deprivation Quintile [*n* (%)]	Quintile 1 - most deprived	114 (6.5)	104 (5.9)	0.93
	Quintile 2	414 (23.6)	414 (23.6)	
	Quintile 3	337 (19.2)	327 (18.6)	
	Quintile 4	390 (22.2)	395 (22.5)	
	Quintile 5 - least deprived	500 (28.5)	515 (29.3)	
GP Practice [*n* (%)]	Practice 1	580 (33.0)	583 (33.2)	1.00
	Practice 2	545 (31.1)	543 (30.9)	
	Practice 3	250 (14.2)	249 (14.2)	
	Practice 4	380 (21.7)	381 (21.7)	

## Results

### Characteristics of patients invited to attend the NHS HC

3511 people were invited to attend an NHS HC across the four practices during the trial period. Table 1 shows the summary characteristics of the 1755 individuals (mean age 53.1) who were sent the control letter and 1756 (mean age 52.8) who were sent the intervention letter. Slightly more women than men were invited. Relatively few (6.2 %) patients from the least deprived quintile were invited compared to the other quintiles. There were no significant differences between the control and intervention group in their demographic characteristics (Table 1).

### Effect of the letter on attendance at the NHS HC

A total of 1102 (31.4 %) individuals attended an NHS HC (29.3 % control invitation letter v 33.5 % intervention invitation letter) (Table 2). This was a 4.2 % absolute difference and a 14.3 % relative difference in attendance by the intervention group. The intervention letter was significantly associated with attendance at an NHS HC (multivariable adjusted odds ratio (AOR) 1.26, 95 % confidence interval (CI) 1.09–1.47, *p* < 0.01). Older age (AOR 1.62, CI 1.50–1.75, *p* < 0.01) and female gender (AOR 1.5, CI 1.29–1.74, *p* < 0.01) were also significantly associated with attendance for an NHS HC. Being in the least deprived quintile was significantly associated with attendance at the health check compared to the most deprived quintile (AOR 1.61, CI 1.14–2.26, *p* < 0.01). Patients from practice 1 were significantly more likely to attend a health check than patients from other practices.

**Table 2:** Numbers invited to, and attending, an NHS HC (with multivariable AORs for attendance)

		Total number invited	Percentage (number) who attended	Multivariable adjusted OR’s for attendance (95 % CI)
Letter	Control	1755	29.3 (514)	1
	Intervention	1756	33.5 (588)	1.26 (1.09–1.47)*
Gender	Male	1678	26.6 (446)	1
	Female	1833	35.8 (656)	1.50 (1.29–1.74)*
Age	10 Years	-	-	1.62 (1.50–1.75)*
Deprivation Quintile	Quintile 1 - most deprived	218	30.7 (67)	1
	Quintile 2	828	26.7 (221)	0.88 (0.62–1.24)
	Quintile 3	664	32.1 (213)	1.24 (0.87–1.75)
	Quintile 4	785	33.9 (266)	1.35 (0.96–1.90)
	Quintile 5 - least deprived	1015	33.0 (335)	1.61 (1.14–2.26)*
GP Practice	Practice 1	1163	38.4 (447)	1
	Practice 2	1088	21.4 (233)	0.33 (0.27–0.41)*
	Practice 3	499	35.9 (179)	0.78 (0.62–0.99)*
	Practice 4	761	31.9 (243)	0.78 (0.64–0.96)*
Total		3511	31.4 (1102)	-

*Note*: One person excluded from the multivariable analysis due to missing deprivation data

*p < 0.01

There was a statistically significant interaction between the letter and the practice (*p* < 0.01). The intervention letter was statistically more effective in practice 4 than in practice 1 (AOR 1.76, CI 1.18–2.64) but other comparisons between practices were not significant. The effects of age, gender and deprivation are unchanged. These results should be interpreted with caution as the study was not powered to detect interaction effects.

## Discussion

Patients receiving the intervention letter were 26 % more likely to attend an NHS HC appointment than patients receiving the control letter. This demonstrates that techniques to simplify information processing, increase the salience and behavioural specificity of desired actions and improve action-planning are all important for increasing uptake of the NHS HCs through letter invitations. Female patients were 50 % more likely to attend than men, and older patients were 62 % more likely to attend with every additional ten years of age. Deprivation was shown to be significant with the most deprived least likely to attend. The least deprived were 61 % more likely to attend. These observed results are all independent of other individual level predictors.

The 4.2 % absolute difference in attendance due to the intervention letters was identical to the effect size observed by Milkman et al. [19] who sent reminders to employees due to attend influenza vaccines in the workplace. These prompts asked employees to record the date and time of their appointment. The same effect was not observed when employees were asked only to record the date of the appointment. Similarly Nickerson and Rogers [21] observed a 4.1 percentage point increase in voter turnout when voters were prompted, via a phone call, to record what time they would vote, where they would be coming from, and what they would be doing beforehand. This phone call also included a self-prediction element asking if the individual intended to vote although self-prediction alone was not found to be effective allowing the authors to conclude the impact was due to the implementation intentions planning prompt. The present research adds evidence to the impact and likely effect size of communications including planning prompts with certain characteristics (date, time and place) in prompting actions related to protective health behaviours. It is possible that this effect could be enhanced by overcoming certain situational barriers which could be constraining behaviour – Kurt Lewin calls these small but critical influences ‘channel factors’. For example, using point of care blood testing to remove the need for more than one appointment. One such example to reduce the hassle factor of health protective behaviours was by Leventhal et al. [30]. They improved student attendance for tetanus vaccines using a pamphlet which shows the university health centre location where the vaccines would take place, times shots were available and a suggestion to plan according to their weekly schedule when they would attend for their vaccination. This resulted in a 22 percentage point increase compared to control.

The findings that females were twice as likely to attend than males is consistent with other findings of gender differences in primary care consultations where rates for females tend to be higher than those for males except in the very young and the very old [31]. Dryden et al. [32] also found that males were less likely to attend general health checks in their narrative scoping review.

There was a significant positive relationship between age and attendance which is reflected in national uptake statistics [31]. Again Dryden et al. [32] found that attenders at general health checks were older than non-attenders. This may be symptomatic of the younger cohort being of working age and finding it hard to attend an appointment within working hours. Older patients may attend primary care more frequently and be more familiar with making and attending appointments due to other screening invitations and eligibility for annual flu vaccination.

While the invitation letter improves the likelihood of a person attending an NHS HC, other factors, such as age and gender, play a larger role in determining whether a person attends an NHS HC. The interaction between practice and letter indicated that effectiveness of the letter varied by practice but this was not consistent across blood test delivery type, which we might have expected. Shah and Oppenheimer highlight that any cue (for example mention of a blood test) that indicates positive or negative associations will be used when evaluating information presented [31]. This is important to consider in the delivery of the NHS HC and thus how it is described in the invitation letter. A larger study is required to determine the effects of deprivation, practice and timing of blood tests. Broader issues such as the timing and venue of the NHS HC, who conducts the NHS HC and practice size, may all be important. Further successes for increasing uptake of the NHS HC could be achieved through more effective targeting of groups known to be less likely to attend a health check for example smokers and men [33]. Ways to achieve this through low cost invitation letters could be through increased personalisation to improve the personal relevance of the invitation. For example including an individuals’ month and year of birth or making specific reference to individual risk factors such as smoking.

### Limitations

It was not possible to truly randomly allocate the participants to the control or intervention letters. Although no differences between intervention and control group characteristics were observed it is possible that this pseudo-randomisation procedure introduced biases of which the researchers are unaware. Arrangements for blood tests differed between practices and it is not possible to know from this study whether this impacted upon the effect of the intervention letter. Further research is needed to explore how different practice characteristics and local delivery procedures affect uptake and how these interact with the invitation process. The study was designed to detect a difference in attendance at an NHS HC between those who were sent the control or intervention invitation letter but was not powered for sub-group analysis. Exploring the effect of enhanced invitation letters with regard to variations in ethnicity and deprivation is an important area for further study. Finally, it was not possible to determine which aspect of the multi-component intervention letter was the driver of improved uptake. Further research would be of value to determine disaggregate and enhance effects, as well as to compare the impact of enhanced letters against other strategies for improving opportunities to attend the NHS HC.

## Conclusions

These findings suggest that making small, low cost changes to the invitation letter using behavioural insights can improve uptake of the NHS HC. However caution should be used when interpreting the generalisability of results to other local areas given the limited number of practices taking part and the large variation between them. Context is an important factor which may influence uptake depending on characteristics of the patient and practice. Given the limited cost associated with making changes to a letter, LAs could implement this letter and closely monitor the effects on uptake.

## Additional files

Additional file 1: Control group letter. (DOC 64 kb) Additional file 2: Intervention group letter. (DOC 66 kb)

## Abbreviations

AOR: adjusted odds ratio
BCT-T V1: Behaviour Change Techniques Taxonomy - Version 1
CI: confidence interval
GP: general practitioner
LA: local authority
NHS HC: National Health Service Health Check
