# Supplementary material for: The effectiveness of an enhanced invitation letter on uptake of National Health Service Health Checks in primary care: a pragmatic quasi-randomised controlled trial
Source: BMC Fam Pract. 2016 Mar 24;17:35. doi: 10.1186/s12875-016-0426-y (PMC4806508; doi:10.1186/s12875-016-0426-y)
Supplement: Additional file 1: — Control group letter. (DOC 64 kb) [file 12875_2016_426_MOESM1_ESM.doc]

Dear Xxxx

We are inviting you to attend your free NHS Health Check.

NHS Health Checks are being offered to people aged between 40 and 74 once every five years.

The check is to assess your risk of developing heart disease, stroke, kidney disease or diabetes. If there are any warning signs, then together we can do something about it.

By taking early action, you can improve your health and prevent the onset of these conditions. There is good evidence for this.

The check should take about 20–30 minutes and is based on straightforward questions and measurements such as age, sex, family history, height, weight and blood pressure. There will also be a simple blood test to measure your cholesterol level.

Following the check, you will receive free personalised advice about what you can do to stay healthy.

Take a look at the enclosed leaflet for more information about the NHS Health Check and how it could benefit you.

Please call the surgery to book your appointment on XXXXXXXX.

Yours sincerely

Dr XXXX
