# Supplementary material for: The effectiveness of an enhanced invitation letter on uptake of National Health Service Health Checks in primary care: a pragmatic quasi-randomised controlled trial
Source: BMC Fam Pract. 2016 Mar 24;17:35. doi: 10.1186/s12875-016-0426-y (PMC4806508; doi:10.1186/s12875-016-0426-y)
Supplement: Additional file 2: — Intervention group letter. (DOC 66 kb) [file 12875_2016_426_MOESM2_ESM.doc]

Dear Xxxx

**You are due to attend your NHS Health Check.**

**Please call us on 0207 222 5555 to book your appointment and record the date and time on the slip below.**

Take a look at the enclosed information about the NHS Health Check and how it would benefit you.

Yours sincerely

Dr XXXXXXXXXXX
